# 4-(4-{[(2-Phenyl­quinazolin-4-yl)­oxy]methyl}-1*H*-1,2,3-triazol-1-yl)butan-1-ol hemihydrate

**DOI:** 10.1107/S1600536811027280

**Published:** 2011-07-13

**Authors:** Abdelaaziz Ouahrouch, Hassan B. Lazrek, Moha Taourirte, Mohamed El Azhari, Mohamed Saadi, Lahcen El Ammari

**Affiliations:** a Laboratoire de Chimie Bio-organique et Macromoléculaire, Faculté des Sciences et Techniques Guéliz, Marrakech, Morocco; b Unité de Chimie Biomoléculaire et Médicinale, Faculté des Sciences Semlalia, Marrakech, Morocco; c Laboratoire de la Matière Condensée et des Nanostructures, Faculté des Sciences et Techniques Guéliz, Marrakech, Morocco; d Laboratoire de Chimie du Solide Appliquée, Faculté des Sciences, Université Mohammed V-Agdal, Avenue Ibn Battouta, BP. 1014, Rabat, Morocco

## Abstract

The title compound, C_21_H_21_N_5_O_2_·0.5H_2_O, has two fused six-membered rings linked to a benzene ring and to a triazole ring, which is connected to a butanol group. The quinazoline ring forms a dihedral angle of 7.88 (8)° with the benzene ring, while the triazole ring is approximately perpendicular to the benzene ring and to the quinazoline system, making dihedral angles of 84.38 (10) and 76.55 (8)°, respectively. The stereochemical arrangement of the butanol chain, with a C—C—C—C torsion angle of 178.34 (19)°, corresponds to an anti­periplanar conformation. However the position of the –OH group is split into two very close [O—O = 0.810(3) Å] positions of equal occupancy. The crystal structure features O—H⋯N and O—H⋯O hydrogen bonds, building an infinite three-dimensional network. The water molecule is located on a half-filled general position.

## Related literature

For details of the synthesis, see: Krim *et al.* (2009 ▶); Mani Chandrika *et al.* (2010 ▶). For the biological activity of quinazolinone derivatives, see: Alvarez *et al.* (1994 ▶); Chan *et al.* (1997 ▶); De Clercq (1997 ▶, 2002 ▶); Dempcy & Skibo (1991 ▶); Gackenheimer *et al.* (1995 ▶). 
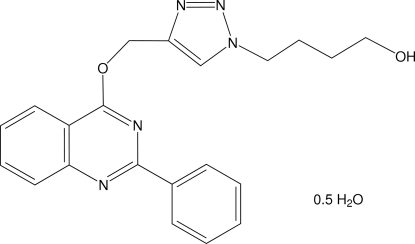


## Experimental

### 

#### Crystal data

C_21_H_21_N_5_O_2_·0.5H_2_O
*M* *_r_* = 384.44
Monoclinic, 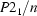

*a* = 11.359 (4) Å
*b* = 7.694 (3) Å
*c* = 22.817 (7) Å
β = 101.111 (16)°
*V* = 1956.9 (12) Å^3^
*Z* = 4
Mo *K*α radiation
μ = 0.09 mm^−1^
*T* = 296 K
0.55 × 0.31 × 0.28 mm

#### Data collection

Bruker X8 APEX Diffractometer
18195 measured reflections
3709 independent reflections
2879 reflections with *I* > 2σ(*I*)
*R*_int_ = 0.028

#### Refinement

*R*[*F* ^2^ > 2σ(*F* ^2^)] = 0.042
*wR*(*F* ^2^) = 0.127
*S* = 1.03
3709 reflections
271 parameters
H-atom parameters constrained
Δρ_max_ = 0.47 e Å^−3^
Δρ_min_ = −0.20 e Å^−3^

### 

Data collection: *APEX2* (Bruker, 2005 ▶); cell refinement: *SAINT* (Bruker, 2005 ▶); data reduction: *SAINT*; program(s) used to solve structure: *SHELXS97* (Sheldrick, 2008 ▶); program(s) used to refine structure: *SHELXL97* (Sheldrick, 2008 ▶); molecular graphics: *ORTEP-3 for Windows* (Farrugia,1997 ▶); software used to prepare material for publication: *WinGX* (Farrugia, 1999 ▶).

## Supplementary Material

Crystal structure: contains datablock(s) I, global. DOI: 10.1107/S1600536811027280/fj2442sup1.cif

Structure factors: contains datablock(s) I. DOI: 10.1107/S1600536811027280/fj2442Isup2.hkl

Supplementary material file. DOI: 10.1107/S1600536811027280/fj2442Isup3.cml

Additional supplementary materials:  crystallographic information; 3D view; checkCIF report

## Figures and Tables

**Table 1:** Hydrogen-bond geometry (Å, °)

*D*—H⋯*A*	*D*—H	H⋯*A*	*D*⋯*A*	*D*—H⋯*A*
O2*B*—H2*B*⋯N1^i^	0.86	2.08	2.935 (3)	170
O2*A*—H2*A*⋯O3*W*^ii^	0.86	1.90	2.757 (4)	176
O3*W*—H3*WA*⋯N3^iii^	0.86	2.12	2.967 (3)	167
O3*W*—H3*WB*⋯N3	0.86	2.00	2.835 (3)	163

Symmetry codes: (i) 
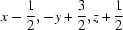
; (ii) 
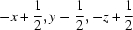
; (iii) 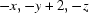.

**Fig. 1.**
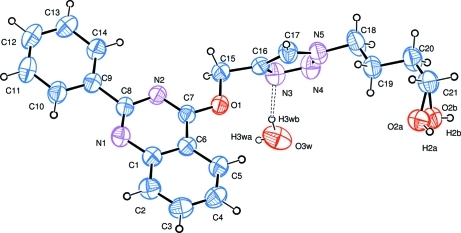
: Plot of the title compound with the atom-labelling scheme. Displacement ellipsoids are drawn at the 50% probability level. H atoms are represented as small circles.

**Fig. 2.**
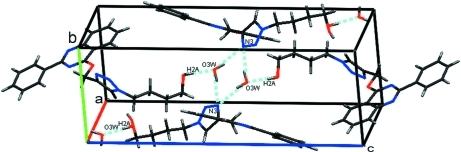
: Tridimensional view of the title compound, showing molecules linked through O—H···N and O–H···O hydrogen bonds (dashed lines).

## supplementary crystallographic information

### Comment

The quinazolinone derivatives are an important class of compounds, as they are present in a large family of products with broad biological activities. For example: anticancers, diuretics, anti-inflammatories, anticonvulsants and antihypertensives (Chan *et al.* (1997); Gackenheimer *et al.* (1995); Dempcy *et al.* (1991)). Also, triazoles associated with various heterocycles are one of the research areas of interesting pharmacological activities, some analogues are used for the treatment of hepatitisC and HIV-1 (De Clercq, (1997); De Clercq, (2002); Alvarez *et al.* (1994)).

The molecule of the title compound is built up from two fused six-membered rings to a phenyl ring and to a five-membered ring which is connected to butanol group as shown in Fig.1. The fused rings are almost planar, with a maximum deviation of -0.0175 (15) Å and -0.0058 (15) Å for C8 and C1 respectively. The dihedral angle between the quinazoline mean plane and the phenyl ring amount to 7.88 (8)°. The triazol ring is approximately perpendicular to the phenyl ring and to the quinazoline system, with a dihedral angles of 84.38 (10)° and 76.55 (8)° respectively. The stereochemical arrangement of the butanol chain with C18—C19—C20—C21 torsion angles in the range of 178.34 (19) ° corresponds to an anti-periplanar conformation.

An intermolecular O—H···N and O—H···O hydrogen bonds, building an infinite three-dimensional network and ensure the cohesion of the crystal structure as schown in Fig.2 and Table 1.

### Experimental

The title compound, 4-(4-((2-phenylquinazolin-4-yloxy)methyl)-1,2,3-triazol -1-yl)butan-1-ol was achieved by cycloaddition of propargylated quinazolinone and azide under microwave conditions with CuI as catalyst and without solvent. The product was obtained with quantitative yield (93%) and short reaction time (Mani Chandrika *et al.* (2010); Krim *et al.* (2009)). The crude product was purified passing through a column packed with silica gel. Crystal suitable for X-ray analysis was obtained by slow evaporation of a methanol / methylene chloride (1:4 *v*/*v*) solution. The melting point is about 371 - 372 K.

### Refinement

The structure is solved by direct method technique and refined by full-matrix least-squares using *SHELXS97* and *SHELXL97* program packages. H atoms were located in a difference map and treated as riding with C—H = 0.97 Å and 0.93 Å for –CH_2_– and aromatic CH respectively. All H atoms with *U*_iso_(H) = 1.2 *U*_eq_ (aromatic, methylene). The O-bound H atom is initially located in a difference map and refined with O—H distance restraints of 0.86 (1). In a the last cycle ther is refined in the riding model approximation with *U*_iso_(H) set to 1.2*U*_eq_(O). In the butanol chain, the OH is statistically distributed on two very close positions with the same occupancy rate and a small atomic displacement parameters and better *R*-factor. The refinement of the occupancy rate of the water molecule led to 0.5 H_2_0 in the unit cell.

### Crystal data

**Table:**

C_21_H_21_N_5_O_2_·0.5H_2_O	*F*(000) = 812
*M**_r_* = 384.44	*D*_x_ = 1.305 Mg m^−^^3^
Monoclinic, *P*2_1_/*n*	Melting point: 371(1) K
Hall symbol: -P 2yn	Mo *K*α radiation, λ = 0.71073 Å
*a* = 11.359 (4) Å	Cell parameters from 3709 reflections
*b* = 7.694 (3) Å	θ = 1.8–25.7°
*c* = 22.817 (7) Å	µ = 0.09 mm^−^^1^
β = 101.111 (16)°	*T* = 296 K
*V* = 1956.9 (12) Å^3^	Block, colourless
*Z* = 4	0.55 × 0.31 × 0.28 mm

### Data collection

**Table:**

Bruker X8 APEX Diffractometer	2879 reflections with *I* > 2σ(*I*)
Radiation source: fine-focus sealed tube	*R*_int_ = 0.028
graphite	θ_max_ = 25.7°, θ_min_ = 1.8°
φ and ω scans	*h* = −13→13
18195 measured reflections	*k* = −9→9
3709 independent reflections	*l* = −27→27

### Refinement

**Table:**

Refinement on *F*^2^	Primary atom site location: structure-invariant direct methods
Least-squares matrix: full	Secondary atom site location: difference Fourier map
*R*[*F*^2^ > 2σ(*F*^2^)] = 0.042	Hydrogen site location: inferred from neighbouring sites
*wR*(*F*^2^) = 0.127	H-atom parameters constrained
*S* = 1.03	*w* = 1/[σ^2^(*F*_o_^2^) + (0.0668*P*)^2^ + 0.4064*P*] where *P* = (*F*_o_^2^ + 2*F*_c_^2^)/3
3709 reflections	(Δ/σ)_max_ < 0.001
271 parameters	Δρ_max_ = 0.47 e Å^−^^3^
0 restraints	Δρ_min_ = −0.20 e Å^−^^3^

### Special details

**Table:**

Geometry. All e.s.d.'s (except the e.s.d. in the dihedral angle between two l.s. planes) are estimated using the full covariance matrix. The cell e.s.d.'s are taken into account individually in the estimation of e.s.d.'s in distances, angles and torsion angles; correlations between e.s.d.'s in cell parameters are only used when they are defined by crystal symmetry. An approximate (isotropic) treatment of cell e.s.d.'s is used for estimating e.s.d.'s involving l.s. planes.
Refinement. Refinement of *F*^2^ against all reflections. The weighted *R*-factor *wR* and goodness of fit *S* are based on *F*^2^, conventional *R*-factors *R* are based on *F*, with *F* set to zero for negative *F*^2^. The threshold expression of *F*^2^ > σ(*F*^2^) is used only for calculating *R*-factors(gt) *etc*. and is not relevant to the choice of reflections for refinement. *R*-factors based on *F*^2^ are statistically about twice as large as those based on *F*, and *R*- factors based on all data will be even larger.

### Fractional atomic coordinates and isotropic or equivalent isotropic displacement parameters (Å^2^)

**Table:**

	*x*	*y*	*z*	*U*_iso_*/*U*_eq_	Occ. (<1)
C1	0.58474 (13)	0.7779 (2)	−0.02725 (7)	0.0442 (4)	
C2	0.69772 (15)	0.8512 (2)	−0.00495 (8)	0.0548 (4)	
H2	0.7529	0.8648	−0.0299	0.066*	
C3	0.72682 (16)	0.9027 (2)	0.05374 (8)	0.0588 (5)	
H3	0.8021	0.9503	0.0682	0.071*	
C4	0.64558 (16)	0.8851 (2)	0.09214 (8)	0.0587 (5)	
H4	0.6669	0.9205	0.1317	0.070*	
C5	0.53502 (15)	0.8160 (2)	0.07146 (7)	0.0515 (4)	
H5	0.4806	0.8044	0.0969	0.062*	
C6	0.50330 (13)	0.7621 (2)	0.01149 (6)	0.0416 (3)	
C7	0.39026 (13)	0.69043 (19)	−0.01484 (6)	0.0410 (3)	
C8	0.45066 (13)	0.6516 (2)	−0.10360 (6)	0.0424 (4)	
C9	0.41915 (14)	0.5786 (2)	−0.16527 (6)	0.0446 (4)	
C10	0.49525 (17)	0.5955 (3)	−0.20563 (8)	0.0595 (5)	
H10	0.5673	0.6552	−0.1945	0.071*	
C11	0.46526 (19)	0.5245 (3)	−0.26255 (8)	0.0692 (5)	
H11	0.5167	0.5390	−0.2893	0.083*	
C12	0.36108 (19)	0.4337 (3)	−0.27949 (8)	0.0634 (5)	
H12	0.3415	0.3852	−0.3175	0.076*	
C13	0.28553 (18)	0.4149 (3)	−0.23962 (8)	0.0630 (5)	
H13	0.2146	0.3525	−0.2507	0.076*	
C14	0.31353 (16)	0.4879 (2)	−0.18315 (7)	0.0543 (4)	
H14	0.2606	0.4756	−0.1570	0.065*	
C15	0.19503 (14)	0.6055 (2)	−0.00380 (7)	0.0499 (4)	
H15A	0.2048	0.4910	−0.0200	0.060*	
H15B	0.1522	0.6786	−0.0355	0.060*	
C16	0.12872 (13)	0.5943 (2)	0.04624 (6)	0.0432 (4)	
C17	0.14267 (15)	0.4820 (2)	0.09333 (7)	0.0537 (4)	
H17	0.1969	0.3905	0.1013	0.064*	
C18	0.04037 (17)	0.4596 (3)	0.18224 (8)	0.0743 (6)	
H18A	0.0219	0.3369	0.1770	0.089*	
H18B	−0.0289	0.5169	0.1926	0.089*	
C19	0.14564 (16)	0.4819 (3)	0.23258 (7)	0.0575 (4)	
H19A	0.1675	0.6038	0.2357	0.069*	
H19B	0.2133	0.4180	0.2232	0.069*	
C20	0.12258 (18)	0.4201 (3)	0.29266 (8)	0.0660 (5)	
H20A	0.0564	0.4864	0.3026	0.079*	
H20B	0.0984	0.2991	0.2892	0.079*	
C21	0.22746 (19)	0.4373 (3)	0.34201 (8)	0.0667 (5)	
H21A	0.2128	0.3960	0.3807	0.080*	
H21B	0.2987	0.3722	0.3308	0.080*	
N1	0.55697 (11)	0.72071 (18)	−0.08592 (6)	0.0481 (3)	
N2	0.36315 (11)	0.63662 (16)	−0.07008 (5)	0.0433 (3)	
N3	0.04075 (12)	0.7060 (2)	0.05220 (6)	0.0568 (4)	
N4	0.00007 (13)	0.6671 (2)	0.10089 (6)	0.0631 (4)	
N5	0.06216 (12)	0.5313 (2)	0.12552 (6)	0.0540 (4)	
O1	0.31014 (9)	0.68013 (15)	0.02114 (4)	0.0493 (3)	
O2A	0.2984 (3)	0.5903 (4)	0.34478 (14)	0.0678 (8)	0.50
H2A	0.3564	0.5896	0.3752	0.081*	0.50
O2B	0.2355 (3)	0.6236 (4)	0.35259 (13)	0.0616 (8)	0.50
H2B	0.1784	0.6580	0.3697	0.074*	0.50
O3W	0.0201 (2)	1.0729 (4)	0.05595 (13)	0.0792 (8)	0.50
H3WA	−0.0015	1.1217	0.0217	0.095*	0.50
H3WB	0.0313	0.9655	0.0481	0.095*	0.50

### Atomic displacement parameters (Å^2^)

**Table:**

	*U*^11^	*U*^22^	*U*^33^	*U*^12^	*U*^13^	*U*^23^
C1	0.0447 (8)	0.0407 (9)	0.0462 (8)	0.0067 (6)	0.0062 (6)	0.0023 (7)
C2	0.0434 (9)	0.0597 (11)	0.0609 (10)	0.0016 (8)	0.0092 (7)	−0.0004 (8)
C3	0.0467 (9)	0.0602 (11)	0.0643 (11)	−0.0007 (8)	−0.0026 (8)	−0.0035 (9)
C4	0.0594 (10)	0.0640 (12)	0.0470 (9)	0.0002 (9)	−0.0041 (8)	−0.0056 (8)
C5	0.0548 (9)	0.0556 (10)	0.0429 (8)	0.0008 (8)	0.0067 (7)	−0.0014 (7)
C6	0.0447 (8)	0.0378 (8)	0.0408 (8)	0.0048 (6)	0.0049 (6)	0.0025 (6)
C7	0.0479 (8)	0.0376 (8)	0.0382 (7)	0.0029 (6)	0.0096 (6)	0.0020 (6)
C8	0.0471 (8)	0.0405 (8)	0.0400 (8)	0.0064 (7)	0.0092 (6)	0.0020 (6)
C9	0.0518 (9)	0.0414 (9)	0.0403 (8)	0.0083 (7)	0.0078 (7)	0.0021 (6)
C10	0.0617 (10)	0.0690 (12)	0.0508 (9)	−0.0010 (9)	0.0181 (8)	−0.0074 (8)
C11	0.0792 (13)	0.0847 (14)	0.0489 (10)	0.0080 (11)	0.0252 (9)	−0.0078 (9)
C12	0.0784 (13)	0.0665 (12)	0.0417 (9)	0.0188 (10)	0.0021 (8)	−0.0097 (8)
C13	0.0666 (11)	0.0667 (12)	0.0516 (10)	0.0004 (9)	0.0010 (8)	−0.0113 (9)
C14	0.0573 (10)	0.0598 (11)	0.0455 (9)	0.0004 (8)	0.0091 (7)	−0.0027 (8)
C15	0.0498 (9)	0.0581 (10)	0.0409 (8)	−0.0092 (8)	0.0066 (7)	−0.0037 (7)
C16	0.0429 (8)	0.0453 (9)	0.0398 (8)	−0.0046 (7)	0.0042 (6)	−0.0028 (6)
C17	0.0592 (10)	0.0535 (10)	0.0498 (9)	0.0066 (8)	0.0137 (8)	0.0050 (8)
C18	0.0621 (11)	0.1113 (18)	0.0518 (10)	−0.0189 (11)	0.0168 (9)	0.0169 (11)
C19	0.0615 (10)	0.0667 (12)	0.0471 (9)	−0.0035 (9)	0.0174 (8)	0.0035 (8)
C20	0.0722 (12)	0.0760 (13)	0.0528 (10)	−0.0031 (10)	0.0194 (9)	0.0104 (9)
C21	0.0831 (13)	0.0668 (13)	0.0500 (10)	−0.0079 (10)	0.0121 (9)	0.0081 (9)
N1	0.0473 (7)	0.0531 (8)	0.0446 (7)	0.0027 (6)	0.0108 (6)	−0.0013 (6)
N2	0.0485 (7)	0.0420 (7)	0.0393 (7)	0.0009 (6)	0.0082 (5)	−0.0011 (5)
N3	0.0527 (8)	0.0646 (10)	0.0523 (8)	0.0077 (7)	0.0081 (6)	0.0070 (7)
N4	0.0480 (8)	0.0879 (12)	0.0544 (8)	0.0095 (8)	0.0121 (7)	0.0051 (8)
N5	0.0463 (7)	0.0714 (10)	0.0442 (7)	−0.0060 (7)	0.0086 (6)	0.0057 (7)
O1	0.0495 (6)	0.0605 (7)	0.0392 (5)	−0.0108 (5)	0.0118 (5)	−0.0065 (5)
O2A	0.076 (2)	0.067 (2)	0.0591 (17)	−0.0163 (18)	0.0112 (16)	−0.0002 (14)
O2B	0.0581 (18)	0.073 (2)	0.0571 (16)	−0.0114 (16)	0.0198 (14)	−0.0072 (13)
O3W	0.0685 (17)	0.0626 (18)	0.098 (2)	0.0057 (13)	−0.0061 (15)	0.0313 (15)

### Geometric parameters (Å, °)

**Table:**

C1—N1	1.387 (2)	C15—O1	1.4409 (19)
C1—C6	1.402 (2)	C15—C16	1.487 (2)
C1—C2	1.404 (2)	C15—H15A	0.9700
C2—C3	1.375 (2)	C15—H15B	0.9700
C2—H2	0.9300	C16—N3	1.345 (2)
C3—C4	1.396 (3)	C16—C17	1.364 (2)
C3—H3	0.9300	C17—N5	1.333 (2)
C4—C5	1.362 (2)	C17—H17	0.9300
C4—H4	0.9300	C18—N5	1.471 (2)
C5—C6	1.409 (2)	C18—C19	1.500 (3)
C5—H5	0.9300	C18—H18A	0.9700
C6—C7	1.420 (2)	C18—H18B	0.9700
C7—N2	1.3058 (19)	C19—C20	1.520 (2)
C7—O1	1.3404 (17)	C19—H19A	0.9700
C8—N1	1.310 (2)	C19—H19B	0.9700
C8—N2	1.3710 (19)	C20—C21	1.479 (3)
C8—C9	1.493 (2)	C20—H20A	0.9700
C9—C14	1.380 (2)	C20—H20B	0.9700
C9—C10	1.385 (2)	C21—O2A	1.421 (4)
C10—C11	1.390 (3)	C21—O2B	1.453 (4)
C10—H10	0.9300	C21—H21A	0.9822
C11—C12	1.364 (3)	C21—H21B	1.0253
C11—H11	0.9300	N3—N4	1.3171 (19)
C12—C13	1.373 (3)	N4—N5	1.324 (2)
C12—H12	0.9300	O2A—H2A	0.8601
C13—C14	1.385 (2)	O2B—H2B	0.8600
C13—H13	0.9300	O3W—H3WA	0.8599
C14—H14	0.9300	O3W—H3WB	0.8599
			
N1—C1—C6	121.74 (14)	N3—C16—C17	107.52 (14)
N1—C1—C2	119.95 (14)	N3—C16—C15	122.48 (14)
C6—C1—C2	118.30 (14)	C17—C16—C15	129.99 (15)
C3—C2—C1	120.02 (16)	N5—C17—C16	105.43 (15)
C3—C2—H2	120.0	N5—C17—H17	127.3
C1—C2—H2	120.0	C16—C17—H17	127.3
C2—C3—C4	121.30 (16)	N5—C18—C19	112.75 (15)
C2—C3—H3	119.4	N5—C18—H18A	109.0
C4—C3—H3	119.4	C19—C18—H18A	109.0
C5—C4—C3	119.83 (16)	N5—C18—H18B	109.0
C5—C4—H4	120.1	C19—C18—H18B	109.0
C3—C4—H4	120.1	H18A—C18—H18B	107.8
C4—C5—C6	119.86 (15)	C18—C19—C20	113.86 (15)
C4—C5—H5	120.1	C18—C19—H19A	108.8
C6—C5—H5	120.1	C20—C19—H19A	108.8
C1—C6—C5	120.69 (15)	C18—C19—H19B	108.8
C1—C6—C7	114.81 (13)	C20—C19—H19B	108.8
C5—C6—C7	124.51 (14)	H19A—C19—H19B	107.7
N2—C7—O1	120.72 (14)	C21—C20—C19	113.89 (16)
N2—C7—C6	123.86 (13)	C21—C20—H20A	108.8
O1—C7—C6	115.42 (12)	C19—C20—H20A	108.8
N1—C8—N2	125.94 (14)	C21—C20—H20B	108.8
N1—C8—C9	118.83 (13)	C19—C20—H20B	108.8
N2—C8—C9	115.22 (13)	H20A—C20—H20B	107.7
C14—C9—C10	118.00 (15)	O2A—C21—C20	118.3 (2)
C14—C9—C8	120.39 (14)	O2B—C21—C20	103.3 (2)
C10—C9—C8	121.60 (15)	O2A—C21—H21A	114.9
C9—C10—C11	120.82 (18)	O2B—C21—H21A	100.7
C9—C10—H10	119.6	C20—C21—H21A	113.8
C11—C10—H10	119.6	O2A—C21—H21B	87.0
C12—C11—C10	120.60 (17)	O2B—C21—H21B	119.7
C12—C11—H11	119.7	C20—C21—H21B	108.9
C10—C11—H11	119.7	H21A—C21—H21B	110.2
C11—C12—C13	119.00 (16)	C8—N1—C1	116.79 (13)
C11—C12—H12	120.5	C7—N2—C8	116.77 (13)
C13—C12—H12	120.5	N4—N3—C16	109.17 (14)
C12—C13—C14	120.86 (18)	N3—N4—N5	107.01 (13)
C12—C13—H13	119.6	N4—N5—C17	110.87 (13)
C14—C13—H13	119.6	N4—N5—C18	120.20 (15)
C9—C14—C13	120.70 (16)	C17—N5—C18	128.88 (17)
C9—C14—H14	119.6	C7—O1—C15	116.95 (11)
C13—C14—H14	119.6	O2B—O2A—C21	75.9 (4)
O1—C15—C16	105.98 (12)	O2B—O2A—H2A	113.3
O1—C15—H15A	110.5	C21—O2A—H2A	111.7
C16—C15—H15A	110.5	C21—O2B—H2A	84.4
O1—C15—H15B	110.5	C21—O2B—H2B	110.5
C16—C15—H15B	110.5	H3WA—O3W—H3WB	105.0
H15A—C15—H15B	108.7		
			
N1—C1—C2—C3	−177.89 (16)	N3—C16—C17—N5	0.08 (18)
C6—C1—C2—C3	1.0 (2)	C15—C16—C17—N5	178.72 (15)
C1—C2—C3—C4	−0.5 (3)	N5—C18—C19—C20	176.04 (18)
C2—C3—C4—C5	−0.1 (3)	C18—C19—C20—C21	178.34 (19)
C3—C4—C5—C6	0.2 (3)	C19—C20—C21—O2A	41.2 (3)
N1—C1—C6—C5	177.93 (15)	C19—C20—C21—O2B	72.5 (2)
C2—C1—C6—C5	−1.0 (2)	N2—C8—N1—C1	2.5 (2)
N1—C1—C6—C7	−2.5 (2)	C9—C8—N1—C1	−176.43 (13)
C2—C1—C6—C7	178.63 (14)	C6—C1—N1—C8	0.2 (2)
C4—C5—C6—C1	0.4 (2)	C2—C1—N1—C8	179.13 (15)
C4—C5—C6—C7	−179.21 (16)	O1—C7—N2—C8	−179.81 (13)
C1—C6—C7—N2	2.3 (2)	C6—C7—N2—C8	0.0 (2)
C5—C6—C7—N2	−178.07 (15)	N1—C8—N2—C7	−2.7 (2)
C1—C6—C7—O1	−177.81 (13)	C9—C8—N2—C7	176.30 (13)
C5—C6—C7—O1	1.8 (2)	C17—C16—N3—N4	−0.03 (19)
N1—C8—C9—C14	172.80 (15)	C15—C16—N3—N4	−178.79 (14)
N2—C8—C9—C14	−6.3 (2)	C16—N3—N4—N5	−0.04 (19)
N1—C8—C9—C10	−5.6 (2)	N3—N4—N5—C17	0.09 (19)
N2—C8—C9—C10	175.30 (15)	N3—N4—N5—C18	177.93 (15)
C14—C9—C10—C11	0.6 (3)	C16—C17—N5—N4	−0.10 (19)
C8—C9—C10—C11	179.04 (17)	C16—C17—N5—C18	−177.71 (16)
C9—C10—C11—C12	−1.2 (3)	C19—C18—N5—N4	−113.8 (2)
C10—C11—C12—C13	0.7 (3)	C19—C18—N5—C17	63.6 (3)
C11—C12—C13—C14	0.5 (3)	N2—C7—O1—C15	0.8 (2)
C10—C9—C14—C13	0.6 (3)	C6—C7—O1—C15	−179.06 (13)
C8—C9—C14—C13	−177.90 (15)	C16—C15—O1—C7	174.82 (12)
C12—C13—C14—C9	−1.1 (3)	C20—C21—O2A—O2B	69.2 (4)
O1—C15—C16—N3	103.78 (17)	C20—C21—O2B—O2A	−122.3 (4)
O1—C15—C16—C17	−74.7 (2)		

### Hydrogen-bond geometry (Å, °)

**Table:**

*D*—H···*A*	*D*—H	H···*A*	*D*···*A*	*D*—H···*A*
O2B—H2B···N1^i^	0.86	2.08	2.935 (3)	170
O2A—H2A···O3W^ii^	0.86	1.90	2.757 (4)	176
O3W—H3WA···N3^iii^	0.86	2.12	2.967 (3)	167
O3W—H3WB···N3	0.86	2.00	2.835 (3)	163

Symmetry codes: (i) *x*−1/2, −*y*+3/2, *z*+1/2; (ii) −*x*+1/2, *y*−1/2, −*z*+1/2; (iii) −*x*, −*y*+2, −*z*.
